# Biosurfactants: Properties and Applications in Drug Delivery, Biotechnology and Ecotoxicology

**DOI:** 10.3390/bioengineering8080115

**Published:** 2021-08-13

**Authors:** Thiago R. Bjerk, Patricia Severino, Sona Jain, Conrado Marques, Amélia M. Silva, Tatiana Pashirova, Eliana B. Souto

**Affiliations:** 1 Institute of Technology and Research (ITP), Av. Murilo Dantas 300, Aracaju 49010-390, Brazil; thiagobjerk@gmail.com (T.R.B.); pattypharma@gmail.com (P.S.); sonajain24@yahoo.com (S.J.); conrado_csl@hotmail.com (C.M.); 2 Industrial Biotechnology Program, University of Tiradentes (UNIT), Av. Murilo Dantas 300, Aracaju 49032-490, Brazil; 3 Department of Biology and Environment, School of Life Sciences and Environment, University of Trás-os-Montes and Alto Douro (UTAD), 5001-801 Vila Real, Portugal; amsilva@utad.pt; 4 Centre for Research and Technology of Agro-Environmental and Biological Sciences (CITAB), University of Trás-os-Montes and Alto Douro (UTAD), 5001-801 Vila Real, Portugal; 5 Arbuzov Institute of Organic and Physical Chemistry, FRC Kazan Scientific Center of Russian Academy of Sciences, Arbuzov St. 8, 420088 Kazan, Russia; tatyana_pashirova@mail.ru; 6 CEB—Centre of Biological Engineering, Campus de Gualtar, University of Minho, 4710-057 Braga, Portugal; 7 Department of Pharmaceutical Technology, Faculty of Pharmacy, University of Coimbra, Pólo das Ciências da Saúde, Azinhaga de Santa Comba, 3000-548 Coimbra, Portugal

**Keywords:** bioengineering, biosurfactants, antimicrobials, drug delivery, polymeric matrices, hydrogels

## Abstract

Surfactants are amphiphilic compounds having hydrophilic and hydrophobic moieties in their structure. They can be of synthetic or of microbial origin, obtained respectively from chemical synthesis or from microorganisms’ activity. A new generation of ecofriendly surfactant molecules or biobased surfactants is increasingly growing, attributed to their versatility of applications. Surfactants can be used as drug delivery systems for a range of molecules given their capacity to create micelles which can promote the encapsulation of bioactives of pharmaceutical interest; besides, these assemblies can also show antimicrobial properties. The advantages of biosurfactants include their high biodegradability profile, low risk of toxicity, production from renewable sources, functionality under extreme pH and temperature conditions, and long-term physicochemical stability. The application potential of these types of polymers is related to their properties enabling them to be processed by emulsification, separation, solubilization, surface (interfacial) tension, and adsorption for the production of a range of drug delivery systems. Biosurfactants have been employed as a drug delivery system to improve the bioavailability of a good number of drugs that exhibit low aqueous solubility. The great potential of these molecules is related to their auto assembly and emulsification capacity. Biosurfactants produced from bacteria are of particular interest due to their antibacterial, antifungal, and antiviral properties with therapeutic and biomedical potential. In this review, we discuss recent advances and perspectives of biosurfactants with antimicrobial properties and how they can be used as structures to develop semisolid hydrogels for drug delivery, in environmental bioremediation, in biotechnology for the reduction of production costs and also their ecotoxicological impact as pesticide alternative.

## 1. Introduction

Surfactants are an important class of chemical compounds widely used in various sectors of modern industry [1,2,3]. The world market is projected to reach USD 52.4 billion by 2025, and it is estimated that the demand for surfactants will increase at a rate of 35% per year. This fact is substantiated in the growing population through growing awareness about the products such as hand sanitizer and anti-inflammatory and emulsification properties due to COVID-19 pandemics [4].

Surfactants are amphiphilic molecules composed of hydrophilic and hydrophobic portions in the same molecule. Surfactants are categorized according to their origin as of synthetic or microbial nature, obtained either from chemical synthesis or produced by microorganisms, respectively. Synthetic surfactants used in various industries, such as the pharmaceutical, medical, agriculture, environmental remediation, and oil industries, have a detrimental role in the environment [5,6].

A concern to look for new alternatives, focusing on ecofriendly [7] and biobased polymeric surfactants [8], is increasingly growing, while biodegradability and sustainability are requirements that led to the development of technologies involving microbial sources. In addition to high biodegradability, they should also be environmentally safe and easily produced. Surfactants obtained from microbial sources also are called biosurfactants. The academic and industrial interest in biosurfactants has increased in recent years due to their diversity, environmentally friendly character, and the possibility of production by fermentation. Additional advantages include their biodegradability, low toxicity, production from renewable sources, functionality under extreme pH and temperature conditions, and stability. These properties enable biosurfactants to be used in emulsification, separation, and solubilization, to reduce surface/interfacial tension and to promote adsorption of bioactives through biological membranes [9,10].

Bacteria, filamentous fungi, or yeast are the microorganisms commonly employed for biosurfactant production. The obtained product can be glycolipids, phospholipids, lipopeptides, fatty acids, saponins, and alkyl polyglycosides. The lipophilic groups can be a protein or a peptide, with hydrophobic parts composed of carbon chains of 10 to 18 carbons or fatty acids. The hydrophilic groups can be amino acids, monosaccharides, disaccharides, or polysaccharides. All biosurfactants under different conditions and other systems show the ability to reduce the surface/interfacial tension of oil/water mixtures [11].

Biosurfactants produced by bacteria are of particular interest due to their antibacterial, antifungal, and antiviral properties with therapeutic and biomedical potential [12]. Their production generally uses pathogenic bacterial species, such as *Pseudomonas* and *Bacillus,* which may be considered a disadvantage attributed to their toxicological risk, requiring the handling of strains in a biosafe environment [11]. Recently, literature has described the use of yeast-like fungi, including *Starmerella bombicola*, and non-pathogenic bacteria such as *sophorolipids* belonging to the genus *Candida bombicola*, to overcome these identified risks [13].

Biosurfactants with lower molecular weight (e.g., glycolipids and low-molecular-weight lipopeptides (LPs) and phospholipids) show industrial potential because of their capacity in reducing surface and interfacial tension. Among glycolipids, those that have a greater interest are trehalolipids, cellobiose lipids, mannosylerythritol lipids, rhamnolipids (derived mostly from *Pseudomonas*), and sophorolipids (SLs) (derived from *Candida* and related species) [14]. Besides, the potential therapeutic uses attributed to these biosurfactants with high molecular weight (such as polysaccharides, lipoproteins, or lipopolysaccharides) include their surface adherence and emulsifier properties.

The antibacterial properties of glycolipids and rhamnolipids are attributed to their permeabilization effect that compromises the integrity of the bacterial plasma membrane. This affects cell surface charge and alters hydrophobicity in a manner similar to the action of synthetic cationic surfactants. They can also make bacteria more susceptible to antimicrobial agents, as they can prevent the formation of biofilms [15].

Sophorolipids produced by yeast show great industrial interest. The molecule consists of the disaccharide, a sophorose, linked to a long chain of hydroxyl fatty chain through a glycosidic bond consisting of hydrophobic characteristics that provide biocidal, cytotoxic, and pro-inflammatory activities, with potential applications in the food, cosmetics, and bioremediation industries [16]. It has been reported that sophorolipid has the capacity to form amphotericin B-loaded niosomal formulation with unique structural characteristics and physicochemical properties, as well as biofilm-breaking functionality and activity [17].

Lipopeptides (LPs) are composed of lipid portions linked to a peptide chain and are also reported to have biological activities, including antimicrobial properties. The most characterized LPs are daptomycin [18] and polymyxin B [19,20,21], lipopeptides microbially derived from antibiotics that have been used in the development of drug delivery systems. Surfactin (SUR), iturin, and fengicin are also among the most well-known LPs and have many potential applications. They accumulate at interfaces showing different polarities, especially oil/water and air/water, and they act as wetting agents on solid surfaces (water/solid). This dynamic process is based on the ability of biosurfactants to reduce surface tension through the placement of their amphiphilic parts in specific areas of the membrane or surface in between the phases. The antimicrobial mechanism is assumed to be LPs polymerization to form transmembrane channels in cells. The antimicrobial activity of biosurfactants is generally quite sensitive to any significant structural change. Chemical synthesis taking into account structure-activity relationships will allow the development of new amphiphiles with improved pharmacological properties, bioavailability, and consequent biodegradability [10,22]. This review summarizes the recent advances and perspectives of biosurfactants, their production from different sources, their physicochemical characterization, and their application as antimicrobial agents, in environmental bioremediation, in pharmaceutics as drug delivery systems, in biotechnology, in the reduction of production costs, and also their ecotoxicological impact.

## 2. Production and Physicochemical Characterization

### 2.1. Synthesis of Biosurfactants of Microbial Origin

Biosurfactants can be obtained from microorganisms’ activity (such as from *Pseudomonas* and *Bacillus*), through enzyme-substrate reactions and fermentation processes, as well as being synthesized extracellularly using biocatalyst enzymes. Both the hydrophobic portion and the hydrophilic portion of the biosurfactants can be synthesized in two independent pathways: both the portions can be substrate-dependent, or one can be synthesized de novo while the other is induced by the substrate [23].

#### 2.1.1. Glycolipid Biosurfactants

Glycolipids are the most common type of biosurfactants. Some of the common glycolipid biosurfactants such as rhamnolipids, trehalolipids, sophorolipids, and mannosylerythritol lipids (MELs) contain mono and disaccharides combined with long-chain aliphatic acids or hydroxy-aliphatic acids [24].

Rhamnolipids are one of the most important glycolipids and are known for their excellent physicochemical properties [23,24]. Rhamnolipids are mainly produced by *Pseudomonas* and *Burkholderia* species. The first step in the production of rhamnolipids includes synthesis of the sugar part containing rhamnose from D-glucose, and the hydrophobic acid part from fatty acids [25]. The enzymes necessary for this first step are usually found in most bacteria, but the specific enzymes needed for the biosynthesis of the rhamnolipids are found almost exclusively in *P. aeruginosa* and *Burkholderia* species. Five different enzymes, RhlA, RhlB, RhlC, RhlG, and RhlI, have been reported to be associated with the production of rhamnolipids in *P. aeruginosa* [26].

Microbial fermentation can result in different kinds of rhamnolipids. Mono-rhamnolipids and di-rhamnolipids differ in the number of rhamnose groups present in the molecular structure. Rhamnolipids also differ with respect to chain length, degree of branching, and degree of unsaturation in the fatty acid chains, all dependent on the environmental and growth conditions [27]. About 60 different rhamnolipid congeners and homologues have been reported [28], using a combination of *Pseudomonas* and other bacterial species. Many *Burkholderia* species have been shown to produce longer alkyl chain rhamnolipids compared to those produced by *P. aeruginosa* [29]. Different substrates, such as alkenes, citrates, glucose, fructose, and olive oil, have also been employed for producing biosurfactants with different properties [27].

A number of studies have been carried out to disclose the best way to produce rhamnolipids, both in terms of safety and cost effectiveness. Engineering *P. aeruginosa* to reduce its pathogenicity is one such way, while another approach involves the expression of the key genes responsible for rhamnolipid production in non-pathogenic strains [30]. The cost of producing rhamnolipids can be reduced by selecting suitable substrates, such as vegetable oils, and optimizing the fermentation process [31]. Sophorolipids can be produced by several non-pathogenic yeast species, genus *Candida* being the most common, and *C. bombicola* and *C. apicola* (containing enzymes necessary for the terminal oxidation of alkanes to generate fatty acids) are the most used species [32]. Sophorolipids can be found in two different forms, the lactonic and the acidic form. Like rhamnolipids, the cost of production of sophorolipids (compared to chemically synthesized surfactants) is very high, which limits their industrial production [33]. Use of oil byproducts or food waste has been suggested as an alternative to reduce the production costs [34].

#### 2.1.2. Lipopeptide Biosurfactants

The biosynthesis of surfactin occurs through a non-ribosomal mechanism catalyzed by surfactin synthetase, a protein complex comprising four enzymatic subunits, among which the subunit SrfD is crucial to initiate the synthesis [35]. Other lipopeptide biosurfactants, such as iturin, lichenysin, and arthrofactin, are also produced by similar synthase complexes [36].

*Bacillus subtilis* is the main bacteria utilized for surfactin production. Genetic engineering of the wild-type strain to improve the low yield has been reported. Wu et al. (2019) modified 53 different genes in *B. subtilis* to reach a yield of around 42% of the theoretical yield [37]. Apart from normal fermentation, surfactin can also be produced through solid state fermentation (SSF), a process in which microorganisms grow on or inside solid substrates or supports, in the absence of free water. Lipopeptide biosurfactants have also been reported to be produced by *Pseudomonas aeruginosa* using renewable resources, such as lubricating oil and peanut oil [38].

#### 2.1.3. High-Molecular-Weight Biosurfactants/Bio-Emulsifiers

Bio-emulsifiers (BE) are high-molecular-weight compounds produced by bacteria, yeast, and fungi. They are synthesized as complex mixtures of heteropolysaccharides, lipopolysaccharides, lipoproteins, and proteins which can be adhered to the cell surface or released [39]. Bio-emulsifiers show a wide variety of physicochemical properties granted by different microbes that produce them [23]. *Acinetobacter* spp. is among the earliest known members producing BEs. Emulsan and Alasan are the best examples of the commercially used bio-emulsifiers produced by *Acinetobacter* spp [40]. Emulsan is a lipoheteropolysaccharide polymer containing D-galactose-amine produced during the stationary phase. Maximum concentration is obtained when culture media containing 12 carbon-based fatty acids are used as the carbon source. Emulsan production is possible with fermentation methods such as batch, chemo-stat, immobilized cell system, and self-cycling fermentation. Other bio-emulsifiers such as mannoprotein have been reported to be produced within the cellular wall of *Saccharomyces* spp. and *Kluyveromyces marxianus* and are released from the cell wall of yeast using pressurized heat treatments [41].

### 2.2. Physicochemical Characterization

Microorganisms are able to produce a wide variety of similar biosurfactants, but with a different bioactivity. In addition to their source, the physicochemical characteristics of these biosurfactants are influenced by production and purification processes. Understanding of these characteristics is important for the correct indication of their industrial application [23]. This section elucidates some important properties of biosurfactants, instrumental for their use as emulsifiers.

#### 2.2.1. Surface and Interfacial Tension

An important feature of a bio-emulsifier is its ability to reduce surface and interfacial tension. This is an essential function of amphiphilic molecules for the formation of kinetically stabilized emulsions. These molecules adsorb on interfaces (air/liquid, liquid/liquid, solid/liquid), replacing water or oil molecules along the interface and reducing intermolecular forces between solvent molecules and surface or interfacial tension [23,24]. Compared to chemical surfactants, biosurfactants were able to decrease the interfacial tension more efficiently [42]. Surfactin is one of the most active surface biosurfactants. Surfactin displays an expressive surface activity from 72 mN/m to 27 ± 2 mN/m [43] and interfacial tension to 3.79 ± 0.27 mN/m and 0.32 ± 0.02 mN/m under harsh physical and chemical conditions [44]. A lipopeptide biosurfactin called arthrofactin produced by *Arthrobacter* sp. strain MIS38 [45] and biosurfactant produced by *Candida lipolytica UCP 0988* [46] showed low surface activity. Such unique surface properties are associated with the more complex chemical structure of biosurfactants. Unlike synthetic surfactants, they do not have a clear distribution of polarity, and also contain branched or ring structures [47]. Lipopeptide surfactin is capable of forming spherical structures to facilitate close packing at interfaces and the formation of structures with a low aggregation number [48]. Unusual surface properties of saponins have been observed. Their behavior was explained by a dense molecular packing at the interface of phases and a strong hydrogen bond between saccharide groups in the interfacial layer [49]. The very compact surface layers formed are denser than those observed in most common amphiphiles. The aforementioned properties of biosurfactants determine their binding mechanisms to biomolecules and cell membranes. A more detailed comparison of properties, namely, surface activity and critical micellization concentration (CMC) values, is presented in the review [50]. In some cases, low CMC values and surface activity can be associated with the presence of impurities in surfactant compositions.

#### 2.2.2. Self-Assembly

**Figure 1:**
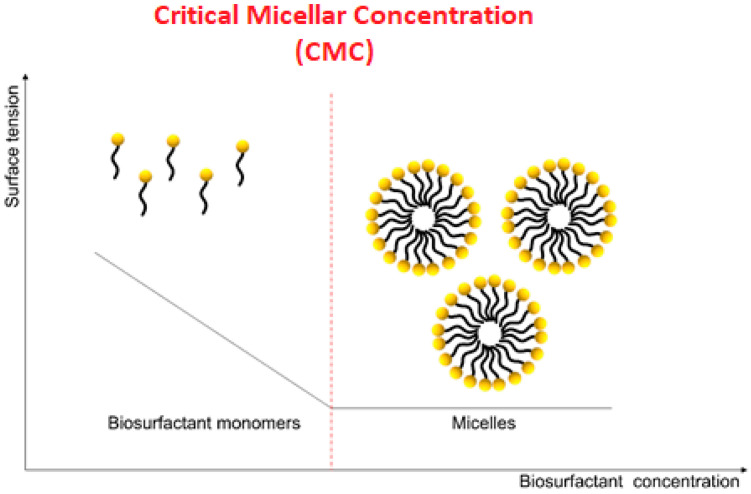
Representation of biosurfactant action. Micelle formation after reaching critical micellar concentration.

Micellization is a balancing process, resulting in thermodynamically stable nanostructures. Surfactants spontaneously form micelles in aqueous solvents at concentrations above the CMC [51]. Figure 1 shows the relationship between surfactant concentration and surface tension. After reaching the CMC, the surfactant monomers are grouped in micelles [52]. Biosurfactants have a tendency to spontaneously self-assemble through hydrophobic effect and weak Van der Waals interactions. The effectiveness of the biosurfactant is defined by its ability to reduce surface tension. The surface tension of the water is 72 mN/m; a good biosurfactant can reduce this value to 30 mN/m [53]. The size or shape of the micelle depends on changes in the concentration of biosurfactant, temperature, pH, pressure, and salts, among others. The repulsive forces of the head groups restrict the amount of associated biosurfactants in the formation of micelles [23]. Increasing the surfactant concentration above the CMC value favors the formation of a greater number of micelles. The formation of micelles with a low aggregation number is characteristic of both rhamnolipids and surfactin. Then, they can be reorganized into bubble structures [54]. While temperature was shown to barely affect surface activity of surfactants, rhamnolipids were transformed into vesicles upon the increase of temperature and decrease of pH [55]. Micelle formation of biosurfactants depends both on the structure of the hydrocarbon chain and on the peptide sequence. The hydrogen bonds between the head groups of biosurfactants lead to the formation of supramolecular structures with different morphologies [56]. The architecture of nanostructures formed by biosurfactants can be nanotubes, spiral, twisted cylindrical nanofibers, and others [57].

#### 2.2.3. Solubilization

Amphiphiles self-assembled in aqueous solutions can solubilize hydrophobic compounds (oil, for example), which preferentially reside in the hydrophobic domains of the amphiphilic nanostructure. The solubility of hydrophobic organic compounds in the presence of biosurfactant depends on the concentration, pH, and the incorporation of additives and salts (electrolytes) that can change the size of micelles [58].

Rhamnolipids can increase the solubilization of hydrophobic compounds by increasing the hydrophobicity of amphiphilic molecule. Biosurfactant molecules have a tendency to form vesicles and micelles with increasing pH, which limits the solubilization of other molecules. The properties of biosurfactants are specific to the substrate, solubilizing or emulsifying different hydrocarbons at different rates [59]. Biosurfactants are more effective solubilizing agents than synthetic surfactants [60]. For example, a biosurfactant obtained from *Rhodococcus erythropolis* HX-2 exhibited a higher solubilization for petroleum and polycyclic aromatic hydrocarbons than synthetic surfactants sodium dodecyl sulfate (SDS), polysorbate (Tween 80), Triton X-100, and rhamnolipid [61]. It is interesting to note that rhamnolipids can solubilize n-alkanes not only at concentrations above CMC [62], but also below CMC [63]. Their solubilization efficiency of n-alkanes is 3–4 order higher below CMC [64]. A synergistic improvement in solubility of polycyclic aromatic hydrocarbons was observed in the case of interaction between two biosurfactants (rhamnolipid and sophorolipid) compared to one glycolipid [65].

#### 2.2.4. Emulsifying Action

Emulsions are kinetically stabilized systems, but without balance. Its structure, stability, and appearance depend on the composition of the liquid phases (oil and water) and of emulsifier (chemical structure and physicochemical properties) and the conditions of the preparation (temperature and pressure) and the process (input energy, mixing time, and the kind of equipment) [66]. The emulsion can be broken down by several mechanisms, including skimming, flocculation coagulation (aggregation of the emulsion droplets), Ostwald maturation, and coalescence. Creaming may happen, caused by the difference in densities between oil and water phases, where the emulsion droplets migrate as a function of the gravitational field, resulting in phase separation [23]. The natural emulsifier must be rapidly adsorbed on the surface of oil droplets, and thus, rapidly reduces the interfacial tension to facilitate droplet breakdown and formation of small droplets [67]. The well-known biosurfactant used as an emulsifier in the food industry is from quillaja saponin extract. The emulsifying activity of the cyclic lipopeptide pseudofactin II produced by *P. fluorescens* BD5 is better than synthetic surfactants Tween 20 and Triton X-100. Pseudofactin II more effectively emulsified aromatic and aliphatic hydrocarbons and some vegetable oils [68]. Rhamnolipids have a greater effect on emulsion droplet size reduction than lecithin and monoglycerides and ensure the thermal stability of emulsion [69].

## 3. Applications

Biosurfactants can have several applications, such as in bioremediation and in food, cosmetics, pharmaceutical, biomedicine, and nanotechnology industries. This may have advantages over their synthetic equivalents as the former are environmentally friendly. Due to their biodegradability and low toxicity, the use of biosurfactants has increased, for example, in the area of biotechnology. One of the most promising areas of application of biosurfactants is in the degradation of hydrocarbons in contaminated water and soil. They can also have a significant impact on the pharmaceutical sector as drug delivery systems [70,71].

According to the work of Marchant et al. [72] and Chakraborty et al. [73], microorganisms use a set of carbon and energy sources for growth. The combination of carbon sources with insoluble substrates favors the diffusion of the intracellular medium and the production of different substances. Moreover, depending on the nature of the carbon source in the culture medium, the synthesis of biosurfactants can be directed to one of the several metabolic pathways involved in this process [74]. In this sense, microorganisms such as bacteria, yeasts, and some filamentous fungi are capable of producing biosurfactants with different molecular structures and surface activities [75,76].

Table 1 shows some of the different residues that have already been used for the production of biosurfactants. Thus, it is possible to observe that several renewable sources and agro-industrial residues containing an excellent source of carbohydrates and lipids can be used as rich sources of carbon and nitrogen for the growth of microorganisms to obtain biosurfactants, in addition to contributing directly to the reduction of the environmental impact caused by these residues [77].

Some examples of residues used for the production of biosurfactants include whey and cheese, animal fat, molasses, glycerol, macerated liquor, residues from the production of olive oil and other extracted vegetable oils, wastewater from cassava processing, and potato, among others [42,78]. Biosurfactants comprise a group of amphipathic molecules with different chemical structures, which are produced by several microorganisms [79]. Besides, these substances, generated mainly from secondary metabolites, play fundamental roles in the survival of their producing microorganisms, contributing in turn to the transport of nutrients and the interactions between the microorganism and the host, as well as acting as biocides [80]. Due to their recognized potential and biological nature, biosurfactants have been the target of numerous researches on their probable therapeutic applications [78,81,82]. As they are of microbial origin and demonstrate a series of interesting characteristics such as low toxicity, pH tolerance, temperature, ionic strength, biodegradability, antimicrobial activity, and emulsifying and demulsifying capacity, these polymers become of great interest in applications in food, cosmetics, and advanced bioremediation processes, including in drug delivery [83].

These agents have several properties suitable for the food industry as emulsifiers in the processing of raw materials, indispensable for food products that require stability content, a characteristic sensory pattern, and longer shelf life, being applied from products derived from meat, milk, and their derivatives. Given their antimicrobial and anti-adhesive properties, these biosurfactants directly contribute to reducing contamination of processed foods [92].

Biosupplements are generally classified as low molecular weight such as glycolipids and lipopeptides and high-molecular-weight biosurfactants which are polysaccharides, proteins, lipoproteins, etc. Generally, low-molecular-weight biosurfactants demonstrate significant active properties due to their relatively simpler structures [93,94]; thus, as shown in Table 2, rhamnolipids (glycolipids) and surfactin (lipopeptide) are among the most studied biosurfactants.

The study by Elshikh et al. [95] demonstrated that the effect of biosurfactants associated with antibiotics as antimicrobial agents presents interesting results. According to the researchers, the combination of nisin and rhamnolipids inhibited thermophilic spores and increased the shelf life of dairy products, while the use of natamycin with rhamnolipid also promoted increased durability of the products, inhibiting the growth of yeasts in industrialized foods. These three associated substances (nisin, natamycin, and rhamnolipids) were also evaluated in some types of cheese and it was observed that there was inhibition of the growth of bacteria and yeasts in these products as well as prolonged shelf life.

These results demonstrate that rhamnolipids have anionic characters mainly due to their carboxylate groups, which, in solution, these groups tend to organize on the cell surface lipid membranes, thus increasing the negative charges and promoting the electrostatic interaction between nisin and anionic membrane, which can result in synergistic effect [96].

### 3.1. Biosurfactants for Environmental Bioremediation

Biosurfactants are promising for environmental bioremediation activities, which include cleaning up oil spills, removing heavy metal contaminants, and treating wastewater. Microorganisms are able to metabolize oil-related compounds, allowing the elimination of hydrophobic pollutants [107]. A critical factor in the biodegradation process is the increase in hydrophobicity on the microbial cell surface caused by biosurfactants. In the biodegradation process, microorganisms use pollutants as carbon and energy sources. These break the hydrocarbon chain, which leads to an immediate loss of amphiphilicity. Finally, the pollutants are transformed into CO_2_, water and minerals [23].

Biosurfactants can improve the hydrocarbon bioremediation, increasing the bioavailability of the substrate for microorganisms or interacting with the cell surface to increase its hydrophobicity, thus allowing an easier association between the hydrophobic substrates themselves and the bacterial cells [99,102].

Several studies have reported the application of microbial surfactants in the process of bioremediation of contaminated soil and wastewater. In the work developed by Patowary et al. (2018) [108], the authors describe the use of rhamnolipid obtained by *Pseudomonas aeruginosa* SR17 for bioremediation of soil contaminated by oil. The degradation of total petroleum hydrocarbon (TPH) in soil containing 6800 ppm and 8500 ppm TPH was evaluated. The efficiency of the rhamnolipid biosurfactant was compared with the synthetic surfactant sodium dodecyl sulfate (SDS). The soil treated with rhamnolipid showed degradation efficiency of 86.1% and 80.5%; by using synthetic surfactant, this value dropped to 70.8% and 68.1%.

Sun et al. (2019) [109] reported the application of biosurfactant glycolipid produced by the strain of *Pseudomonas aeruginosa* S5 isolated from coking effluent for in situ remediation. The inoculation of the strain in the coke wastewater promoted the biodegradation of polycyclic aromatic hydrocarbons, reducing 44% in 15 days in the sludge.

Zhou et al. (2020) [110] evaluated the effects of the addition of the lipopeptide produced by *Acinetobacter* sp. isolated from hydraulic fracturing flowback and produced (HF-FPW). The authors observed that inoculation of the bacterial strain in HF-FPW increased the activity and growth of *Pseudomonas* sp. and *Rhizobium* sp., known for their hydrocarbon degradation capacity, achieving a reduction of 94% and 77% for n-alkanes and polycyclic aromatic hydrocarbons in 7 days. Recently, Swati et al. (2020) [111] showed that the biosurfactant produced by the strain *Pseudomonas* sp. ISTPY2, isolated from the Ghazipur landfill, has a degradation efficiency of pyrene present in high concentration in the soil microcosm, reaching a removal efficiency of 94% in 10 days.

Tang et al. (2018) [112] evaluated the application of rhamnolipid, saponin, and sophorolipid biosurfactants to increase the removal of heavy metals Cu, Zn, Cr, Pb, Ni, Mn, Fe, and Hg from the sludge through electrokinetic treatment. The results showed that three biosurfactants can effectively increase the removal of heavy metals from the sludge, showing chelating and binding capacity under acidic conditions.

Recently, Sun et al. (2021) [113] evaluated the application of biosurfactant produced by *Pseudomonas* sp. CQ2 isolated from the Chongqing oilfield (China) for bioremediation of heavy metals in contaminated soil. Removal efficiency of 78.7, 65.7, and 56.9% was obtained for Cd, Cu, and Pb, respectively, values higher than those obtained using chemical surfactants.

### 3.2. Pharmaceutical Applications of Biosurfactants

Biosurfactants can assume an immense variety of functions in the pharmaceutical industry, since they have antimicrobial, anti-adhesive, antiviral, anticancer, spermicidal, hemolytic, anti-inflammatory, and immunomodulatory activities [24,114,115,116,117,118,119].

Antimicrobial application is one of the most desired uses described in pharmaceutical literature. Surfactin has several interesting properties, including antimicrobial, antiviral, antitumor, hypocholesterolemic, anti-adhesive, insecticide, apoptotic, and hemolytic action. Among other functions, surfactin inhibits the fibrin clotting process and has antitumor activity against Ehrlich’s ascites carcinoma cells, inhibiting cyclic adenosine 3′, 5′-monophosphate phosphodiesterase, and antifungal activities [23,82].

Among the studies, lipopeptide biosurfactants showed in vitro antimicrobial, antibiofilm, and cytotoxic effects. The biosurfactant was produced by *Acinetobacter junii* (AjL), exhibiting inhibition against *Candida utilis* and becoming a potential new drug [120]. In this way, Fernandes et al. [121] used kitchen waste oil as *Wickerhamomyces anomalus* CCMA 0358 substrate to produce a biosurfactant with larvicide activity, *Aedes aegypti* larvae. The biomolecule also assesses activity against bacterial (*Bacillus cereus, Salmonella enteritidis, Staphylococcus aureus,* and *Escherichia coli*) and fungal strains (*Aspergillus, Cercospora, Colletotrichum,* and *Fusarium*). The main advantages concern the low cost of production and ease of scale up process.

Jiang et al., 2019 [122] produced biosurfactants using *Lactobacillus helveticus* strains showing anti-adhesive and inhibiting effects on biofilm formation and this also was observed in studies carried out by Ashitha et al. (2020) [123] using *Burkholderia* sp. WYAT7 strains to obtain biosurfactant with antibacterial activity against *Pseudomonas aeruginosa* (MTCC 2453), *Escherichia coli* (MTCC 1610), *Salmonella paratyphi*, and *Bacillus subtilis* strains.

Moreover, biosurfactants are promising for cosmetic applications. According to recent publications, they have lower toxicity, offering dermal compatibility and moisturizing effects [124]. Ferreira et al., 2017 [125] described a demand for green cosmetics to replace petroleum derivates. The researchers developed a biosurfactant originating from *Lactobacillus paracasei* by oil-in-water (o/w) emulsification. These results were compared to the traditional surfactant, sodium dodecyl sulphate (SDS), used in the cosmetics industry, and they obtained similar results. No toxicity effect was observed in fibroblasts, otherwise SDS showed a strong inhibitory effect.

Biosurfactants have been employed as a drug delivery system (DDS) to improve the oral bioavailability of a large number of drugs that exhibit low aqueous solubility, and this has been a major challenge in the field of pharmaceutical sciences. The great potential of these molecules concerns in auto assembly and emulsification [126]. Hereby, some strategies, such as microemulsion drug delivery systems (MDDS), have been adopted to develop delivery systems capable of improving the oral bioavailability of hydrophobic drugs. They contain lipids, surfactants, cosurfactants, and/or cosolvents, and are generally globular in shape [127]. Currently, MDDS are being formulated for use through various routes of administration, such as oral, nasal, ocular, topical, and intravenous [128].

Hydrogels incorporating antimicrobial biosurfactants have been suggested as wound healing systems against skin drug-resistant infections [129]. Hydrogels obtained from poly (vinyl alcohol) (PVA), polyethylene oxide (PEO), and poly (acrylic acid) (PAA) polymers can be used as a semisolid vehicle for topical products containing antimicrobial surfactants. Examples of commercially available products containing antimicrobial biosurfactants are the moisturizer Kanebo (Kanebo Cosmetics, Tokyo, Japan), the facial cleaner Sopholiance (Givaudan Active Beauty, Paris, France), and the body moisturizer Relipidium (BASF, Monheim, Germany) [130].

### 3.3. Biotechnological Applications of Biosurfactants

In spite of the ever-increasing demand, the commercial production of biosurfactants is still a challenge due to high raw material costs and high processing costs together with low output. Thus, one of the current research challenges includes an increase in the yield, keeping the cost of raw materials as low as possible. Several studies have aimed to optimize the biosurfactant production process by changing the variables such as carbon and nitrogen sources, amount of oxygen, temperature, and pH that influence the type and amount of biosurfactant produced by a microorganism. However, recently, many studies have focused on biosurfactant production using renewable substrates [131], which will be the focus of this section.

Various research groups around the globe have explored the use of inexpensive alternative sources (such as agroindustrial waste) to produce different biosurfactants. The use of industrial/house byproduct/waste as raw material brings down the production costs, and at the same time reduces the environmental damage by reducing their accumulation. A large variety of waste and byproducts have been described in the literature for biosurfactants production, including oil processing waste, starch waste, sugar industry waste, fruit and vegetable waste, distillery waste, and animal fat, some of which have been listed in Table 1.

Industrial starch production using crops such as corn, rice, cassava, wheat, and potato generate high amounts of wastewater, rich in starch, which can be used as a substrate for the production of biosurfactants. Fox and Bala (2000) [132] described the use of potato substrates for producing surfactants from *B. subtilis* and reported a surface tension of 28.3 mN/m in solid medium and a CMC of 100 mg/L, using 60 g/L of potato substrate in the cultivation media. Minucelli et al. (2017) [133] reported the use of chicken fat, sunflower oil, sugarcane molasses, sugarcane juice, sucrose, or glucose for the production of sophorolipids by *C. bombicola*. About 39.8 g/L of sophorolipids were produced using 75 g/L chicken fat, 77.5 g/L glucose, and 2.5 g/L yeast extract, giving a surface tension of 35 mN/m and a CMC of 65 mg/L. Similarly, Chaves-Martins and Guimarães-Martins (2018) [134] reported the use of different industrial waste (sugarcane bagasse, fish waste, crude glycerol, and petroleum sludge from storage tanks) for biosurfactant production using *Corynebacterium*. Efficient biosurfactant production was achieved with the use of 3% sugarcane bagasse and 3% fish residue as a carbon source with ST values of 27.8 mN/m and 33.9 mN from sugarcane bagasse and fish residue, respectively.

Compared to synthetic surfactants (priced at approximately USD 2/ kg, Santos et al. (2016) [74]), the price of biosurfactants can range from anywhere between USD 20/kg and USD 1250/kg. AGAE Technologies, LLC (USA, www.agaetech.com (accessed date: 19 May 2021)) lists a price of USD 1250/kg approximately for rhamnolipids, while the surfactin, iturin, and fengycin marketed by Sigma-Aldrich Co. LLC, USA are listed at USD 206/10 mg, 527/5 mg, and 530/5 mg, respectively (www.sigmaaldrich.com (accessed date: 19 May 2021)).

Compared to these, Soares da Silva et al. (2018) [135] described production of glycolipid (40.5 g/L) (produced in a 50 L fermenter) via *P. cepacia* with canola frying oil at an estimated price of USD 20/kg. Similarly, Ashby et al. (2013) [136] reported sophorolipid production at a cost of USD 2.54/kg, using glucose and high oleic sunflower oil. As raw materials constitute about 50% of the overall biosurfactant production (Rufino et al., 2014), the use of cheaper agroindustrial waste and low-cost renewable substrates can substantially reduce the production costs. The use of agroindustrial waste is thus a potential approach for reducing production costs that could make biosurfactants economically viable and commercially competitive with synthetic surfactants.

## 4. Ecotoxicology

Every year, plantations around the world are damaged by phytopathogens, which leads to great economic loss. Currently, the most common way to combat this problem is the use of pesticides, which is a concern for environmentalists. Many biosurfactants produced by microorganisms are being studied for their potential to inhibit the growth of phytopathogens by presenting antimicrobial and antibiofilm properties [137,138]. The plant-associated microbiome produces a structurally diverse group of compounds with hydrophilic and hydrophobic portions, which exhibit biosurfactant activity. These can be lipopeptides, glycolipids, phospholipids, polysaccharides, neutral lipids, and fatty acids [138]. These biosurfactants are an ecofriendly alternative compared to synthetic surfactants due to their lower toxicity and biodegradable properties [23].

**Figure 2:**
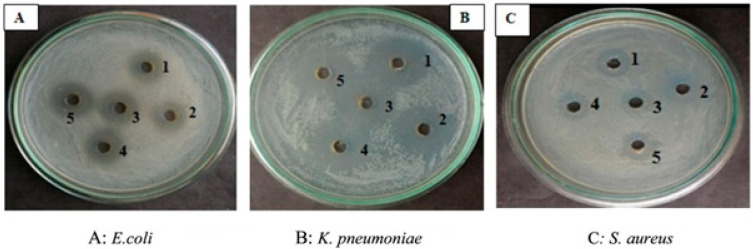
Antibacterial activity of extracted lipopeptide. (**A**): *Escherichia coli*; (**B**): *Klebsiella pneumoniae*; (**C**): *Staphylococcus aureus*. Here, 1 to 5 are different concentrations of extracted lipopeptide from 10 to 50 μg/mL (with the permission after [139]).

Chittepu (2019) [139] isolated and characterized the bacterium *Bacillus pseudomycoides* OR 1 that produces peanut pie dump sites. The lipopeptide from *B. pseudomycoides* OR 1 at a concentration of 50 μg/mL demonstrated greater antibacterial activity against *E. coli*, *K. pneumoniae,* and *S. aureus*, respectively (Figure 2).

In a recent study, Ohadi et al. (2020) [116] evaluated in vitro antimicrobial and antibiofilm effects of the lipopeptide produced by *Acinetobacter junii*. The biosurfactant showed non-selective activity against Gram-positive and Gram-negative bacterial strains. It was found that the minimum inhibitory concentration values for *C. albicans and C. utilis* were smaller than the standard fluconazole antifungal. The authors also highlighted a reduction in the biofilm formation of *Staphylococcus aureus*, *Proteus mirabilis*, and *Pseudomonas aeruginosa* by 52%, 31%, and 70%, respectively, in the concentration of 2500 μg/mL of biosurfactant.

Ashitha et al. (2020) [140] identified the glycolipid biosurfactant produced by *Burkholderia* sp. WYAT7 isolated from the medicinal plant *Artemisia nilagirica* (Clarke) Pamp and evaluated its antibacterial and antibiofilm potential. The biosurfactant exhibited antibacterial activity against bacterial pathogens, such as *P. aeruginosa* (MTCC 2453), *E. coli* (MTCC 1610), *S. paratyphi*, and *B. subtilis*. Glycolipid at a concentration of 2 mg/mL showed 79% antibiofilm activity against *S. aureus* (MTCC 1430).

## 5. Conclusions

Numerous scientific publications, reporting the especial properties of biosurfactants (e.g., antimicrobial, emulsifying, and anti-adhesive), produced either by chemical synthesis or by microorganisms’ activity, suggest the potential applications of such compounds in a range of industries. New biosurfactants (glycolipids, surfactin, and high-molecular-weight biosurfactants) produced by microorganisms (bacteria, yeasts, fungi) that may contribute to the detection of different molecules in terms of structure and physicochemical characteristics, are increasingly enhancing antimicrobial aspects, precisely in the area of food and medicine safety, defense of plants and animals, as well as in the control and treatment of diseases. Their amphiphilic nature and capacity for self-assembly open perspectives in the development of new formulations with antimicrobial properties for the delivery of bioactives of pharmaceutical interest. For topical application, these drug delivery systems can be formulated in semisolid hydrogels that can be exploited against skin drug-resistant infections and for wound treatment. Several other examples on the use of biosurfactants to reduce the costs of biotechnological products have been reported, such as the use of surfactants from *B. subtilis* to improve cultivation media. With respect to the ecotoxicological impact, due to their antimicrobial and antibiofilm properties, several biosurfactants produced from microorganisms (e.g., lipopeptides, glycolipids, phospholipids, polysaccharides, neutral lipids, and fatty acids) have found utility in inhibiting the growth of phytopathogens. It is thus expected that biosurfactants are to be considered a potential alternative with several industrial applications.

## Figures and Tables

**Table 1:** Different residues used in the production of biosurfactants by different microorganisms.

Waste Products	Producing Microorganism	Type of Biosurfactant	Ref.
Whey	*Pseudomonas aeruginosa* BS2	Rhamnolipid	[84,85]
	*Bacillus* spp.	Lipopeptides	[86]
Molasses	*Pseudomonas aeruginosa* GS3	Rhamnolipid	[87]
	*Bacillus* spp.	Lipopeptides	[35,88]
	*Starmerella bombicola NBRC 10243*	Lipopeptides Sophorolipids	[89] [13,90]
Potato processing effluents and cassava wastewater	*Bacillus subtilis*	Lipopeptides	[31]
Frying oil	*Pseudomonas aeruginosa* 47T2 4	Rhamnolipid	[91]
Corn steep liquor	*Aureobasidium thailandense*	Glycolipid	[77]
	*Candida lipolytica*		[46]
Refinery oil waste	Yeast	Glycolipid	[33,77,89]

**Table 2:** Biosurfactants and their applications.

Type of Biosurfactant	Microorganism	Application	Reference
Rhamnolipids	*Pseudomonas aeruginosa, Pseudomonas putida*	Bioremediation	[97]
	*Pseudomonas chlororaphis*	Biocontrol Agent	[98]
	*Renibacterium salmoninarum*	Bioremediation	[99]
Sophorolipids	*Candida bombicola, Candida apicola*	Emulsifier	[100]
Glycolipids	*Rhodococcus* spp.	Bioremediation	[101]
	*Tsukamurella* sp., *Arthrobacter* sp.	Antimicrobial agent	[102,103]
Manosileritritol lipids	*Candida antartica*	Anti-inflammatory secretion inhibitor and RBL-2H3 cell mediators	[76]
Surfactin	*Kurtzmanomyces* sp	Biomedical application	[80,104]
Lipopeptides	*Bacillus subtilis*	Bacterial growth inhibitionBiomedical application	[105]
Lichenisina	*Bacillus licheniformis*	Antimicrobial activityHemolytic and chelating agent	[86]
Glycolipoprotein	*Aspergillus niger*	Antimicrobial activity	[106]

## Data Availability

Not applicable.
